# Observed and expected serious adverse event rates in randomised clinical trials for hypertension: an observational study comparing trials that do and do not focus on older people

**DOI:** 10.1016/S2666-7568(21)00092-1

**Published:** 2021-07

**Authors:** Peter Hanlon, Neave Corcoran, Guy Rughani, Anoop S V Shah, Frances S Mair, Bruce Guthrie, Joanne P Renton, David A McAllister

**Affiliations:** a Public Health, Institute of Health and Wellbeing, University of Glasgow, Glasgow, UK; b General Practice and Primary Care, Institute of Health and Wellbeing, University of Glasgow, Glasgow, UK; c Department of Non-communicable Disease Epidemiology, London School of Hygiene & Tropical Medicine, London, UK; d Usher Institute for Population Health Sciences, University of Edinburgh, Edinburgh, UK

## Abstract

**Background:**

Representativeness of antihypertensive drug trials is uncertain, as many trials recruit few or no older people. Some trials specifically recruit older participants to address this. Here, we assess the representativeness of trials focusing on older people by comparing the rates of serious adverse events in these trials with the rates in trials of a general adult population (ie, standard trials), and comparing these findings to the rate of hospitalisations and deaths in people with hypertension starting a similar treatment in routine clinical practice.

**Methods:**

For this observational study, we identified randomised controlled trials (phase 2/3, 3, or 4) of renin-angiotensin-aldosterone system (RAAS) drugs for hypertension registered from 1999 onwards with ClinicalTrials.gov. Serious adverse events are routinely included in trial reports and are predominantly accounted for by all-cause hospitalisations and deaths. We compared serious adverse event rates in older-people trials (minimum inclusion age ≥60 years) and standard trials (minimum inclusion age <60 years) using Poisson regression models adjusted for trial characteristics (drug type, comparison type, phase, and outcome type). We identified a community cohort of 56 036 adults with hypertension commencing similar drugs to obtain an expected rate of emergency or urgent hospitalisations or deaths, and compared this rate to observed serious adverse event rates in each trial, adjusted for age and sex. For standard trials and for older-people trials, we calculated the standardised ratio of the expected to the observed rate of serious adverse events using Poisson regression models.

**Findings:**

We included 110 trials, of which 11 (10%) were older-people trials and 99 (90%) were standard trials. Older-people trials had a higher rate of serious adverse events than did standard trials (median events per person per year 0·18 [IQR 0·12–0·29] *vs* 0·11 [0·08–0·18]; adjusted incidence rate ratio 1·76 [95% CI 1·01–3·03]). The hospitalisation and death rate in the community for those taking RAAS antihypertensives was much greater than the rate of serious adverse events reported in standard trials (standardised ratio [SR] 4·23, 95% CI 3·51–5·09) and older-people trials (4·76, 2·89–7·86), adjusting for age and sex. The magnitude of risk increase for serious adverse events in community patients taking RAAS did not differ when comparing older-people and standard trials (ratio of SRs 1·13, 95% CI 0·66–1·92).

**Interpretation:**

Trials report substantially fewer serious adverse events than expected from rates of hospitalisations and deaths among similar-aged people receiving equivalent treatments in the community. Serious adverse event rates might be a useful metric to assess trial representativeness. Clinicians should be cautious when applying trial recommendations to older people, even when trials focus on older participants.

**Funding:**

Wellcome Trust, Medical Research Council.

## Introduction

Hypertension is a common and important modifiable risk factor for major cardiovascular disease, and is associated with age, with more than 75% of people older than 80 years diagnosed with hypertension.^1^ There is uncertainty, however, about how hypertension should best be managed in older people.^2^ The risk of cardiovascular disease associated with hypertension might reduce as people age,^3^ particularly in people living with frailty.^4^ Furthermore, antihypertensive treatment presents a range of potential risks that might disproportionately affect older people.

Although randomised controlled trials provide the least-biased estimates of treatment efficacy, there are concerns that trial participants are often not representative of people treated for hypertension in routine clinical practice.^5^ Specifically, older people are often excluded from trials.^6^ This can occur directly, through age-based exclusion criteria, or indirectly, through other exclusion criteria that disproportionately exclude older people (eg, comorbidity or co-prescribing), as well as through the trial recruitment process (eg, through increased demands of participating in a trial, or patient or clinician preferences).^6^, ^7^ To address this problem and provide evidence to guide treatments for older people, some trials have focused explicitly on older people.^8^, ^9^ However, such trials often only enrol a fraction of those invited to participate.^10^ Consequently, it remains unclear whether conducting trials specifically focused on older people is sufficient to overcome the difficulties in applying trial evidence to older people encountered in routine clinical practice.

**Research in context**

**Evidence before this study**

We searched MEDLINE from inception to Nov 5, 2020, without language restrictions, using the terms “hypertension” and “trials”, and (“representative*” or “serious adverse events”) for studies assessing representativeness of hypertension trial populations or assessing the rates of serious adverse events in hypertension trials. Four studies, including 24 different trials, assessed the representativeness of hypertension trials by applying trial exclusion criteria to people with hypertension in routine clinical practice. The proportion of people who were ineligible for trials was between 50% and 100% in most cases. This was true of trials specifically focusing on older adults (eg, the HYVET, SPRINT and OPTiMISE trials) in which polypharmacy, multimorbidity, and frailty were associated with ineligibility. This suggests that trial participants are likely to be healthier overall than people treated in the community. Previous studies have not directly compared health-related outcomes of trial participants with real-world populations. Older adults have been shown to have higher rates, and a greater diversity, of adverse events in the trial setting. However, we did not identify any previous studies that systematically assessed rates of serious adverse events in hypertension trials, that compared serious adverse events in trials focusing on older people with other trials, or that compared serious adverse events in the trial population with similar events in community populations.

**Added value of this study**

After systematically identifying hypertension trials of drugs acting on the renin-angiotensin-aldosterone system, we showed that trials focusing on older people had a significantly higher rate of serious adverse events than comparator trials that did not focus specifically on older people (ie, standard trials). As would be expected, this suggests that trials focusing on older people recruited people with a greater risk of adverse health outcomes than trials including all ages. However, the rate of all-cause hospitalisations and deaths (which, by definition, would be serious adverse events in trial populations) among people with hypertension treated in the community was on average four times higher than the serious adverse event rate in the trials, after adjusting for age and sex. This difference in rates was similar for standard trials and trials focusing on older people. Therefore, despite having a higher risk of serious adverse events than in standard trials, people included in hypertension trials focused on older people have a considerably lower incidence of adverse health outcomes than people of a similar age receiving similar treatment in the community. This shows that there are clinically meaningful differences between trial populations and people treated for hypertension in the community. Furthermore, where serious adverse event rates in trials are lower than expected, this should prompt careful consideration of trial exclusion criteria and population characteristics when assessing representativeness and applicability.

**Implications of all the available evidence**

Our findings show that people in hypertension trials experience substantially lower rates of adverse health outcomes than people with hypertension treated with similar drugs in the community. This adds weight to the body of evidence showing that hypertension trials are under-representative of their target populations. However, our findings also add nuance to this notion, as trials focusing on older people have a significantly higher rate of serious adverse events than do standard trials. Therefore, trials focusing on older people do, at least in part, reflect the increased risk of adverse outcomes seen in older populations. Trials focusing on older people therefore have an important role in informing treatment decisions in older people, but should be viewed with caution as, like standard trials, they are not representative of community populations. Our findings also indicate that serious adverse event rates should be considered as a novel metric with which to assess the representativeness of trial populations, through comparison with the incidence of similar events in routine clinical care. Such an approach could facilitate more direct quantification of the consequences of trial under-representativeness; however, this would require consistent and complete recording and reporting of serious adverse events as well as reliable estimates of event rates in the community.

Older people have a greater risk of adverse health outcomes in routine care settings and in trials.^11^ This is likely to be driven by characteristics such as frailty, multimorbidity (increasing the risk of drug–disease interactions), polypharmacy (increasing the risk of drug–drug interactions), and decreased kidney and liver function. All these characteristics are more common in older age, associated with poor health outcomes, and often under-represented within trials.^12^, ^13^, ^14^, ^15^, ^16^

Previous studies assessing trial representativeness have tended to apply trial exclusion criteria to population samples derived from routine health-care data or disease registries, concluding that many people living with long-term conditions would be ineligible for trials.^5^, ^6^, ^10^, ^17^ However, such an approach does not directly assess the health outcomes in trial participants compared with those receiving routine care. An alternative approach is to analyse serious adverse events. Serious adverse events in a trial setting are events that are either life threatening, lead to death, cause or prolong hospitalisation, result in serious or lasting impairment or disability, or cause a birth defect.^18^ Among serious adverse events, hospitalisations and deaths are the most common. Regulatory bodies require that trial sponsors record and report all serious adverse events,^19^ with recording also part of the CONSORT statement for the publication of trial findings.^20^ Importantly, serious adverse events are required to be reported irrespective of the suspected cause, for both treatment and control arms. Therefore, serious adverse events should provide a reliable measure of the rate of adverse health outcomes (particularly resulting in hospitalisation and death) within a trial population. Indeed, if a trial were perfectly representative, we would expect the serious adverse event rate of that trial to be similar to hospitalisation and death rates among the target population with the same condition, to whom the trial results are intended to apply. We would also expect trials involving older people to have higher serious adverse event rates than trials for the same indication recruiting a more general adult population.

The aim of this Article is to compare the rates of serious adverse events in trials of older people with the rates found in trials not focusing specifically on older people (which we will call standard trials), and to compare rates of serious adverse events in each trial to the rate of serious adverse events (ie, hospitalisations and deaths) in people with hypertension starting a similar treatment in routine clinical practice, adjusting for age and sex. As an exemplar, here we focus on drugs to treat hypertension acting on the renin-angiotensin-aldosterone system (RAAS). RAAS drugs were chosen since they are commonly used to treat hypertension, including in older people. There are also concerns that older people are under-represented in RAAS trials.^5^, ^17^, ^21^ Furthermore, associated risks of RAAS drugs such as renal dysfunction, orthostatic hypotension, syncope, and polypharmacy are likely to be greater in older people.^22^

## Methods

### Study design and participants

This observational study compares serious adverse event rates in registered randomised controlled trials of RAAS drugs to treat hypertension with hospitalisation and death rates in a community sample of adults with hypertension who were initiated on RAAS drugs. Trials were identified from an extraction on Aug 1, 2017, of all clinical trials registered at ClinicalTrials.gov (a registry of clinical trials from across the world managed by the US National Institutes of Health), to which we had applied the WHO Anatomic Therapeutic Chemical (ATC) drug classes for all interventions.^12^ To be eligible, trials had to be registered from 1999 onwards, be phase 2/3, 3, or 4, have eligibility criteria published in English, and be evaluating RAAS drugs for the treatment of hypertension. We included trials in two stages. First, we identified all trials with a minimum inclusion age of 60 years or older and defined these as trials of older people. We reviewed these to identify the drugs and indications for which such trials were commonly undertaken. Second, we obtained, as a comparator group, all trials for the same indications and drugs with a minimum inclusion age of less than 60 years (ie, standard trials). We included single-centre or multicentre trials undertaken in any country, with published or unpublished results.

The community comparison sample was identified using the Secure Anonymised Information Linkage (SAIL) databank. SAIL collects routine health-care data (including primary care diagnostic codes and prescriptions, with linked hospital and mortality data) from participating practices in Wales (covering approximately 70% of the Welsh population). SAIL participants are representative of the Welsh population in terms of age, sex, and socioeconomic status. We identified adult participants with a previous diagnostic code for hypertension in primary care who were prescribed a RAAS drug for the first time between Jan 1, 2011, and Dec 31, 2015. We excluded participants who registered with a SAIL practice less than 12 months before starting treatment with the RAAS drug. We also excluded people with any coded myocardial infarction or stroke occurring in the 12 months before treatment initiation, as these people were unlikely to be receiving the RAAS drug solely to treat hypertension and so were likely to have higher rates of hospitalisations and deaths.

### Measures

We extracted the following information on included trials from ClinicalTrials.gov, clinical trial reports, and published papers: baseline characteristics of the trial participants (age, sex, body-mass index), number of trial participants, trial phase, trial drug, comparison treatment, outcomes, follow-up times, and the occurrence of serious adverse events (total number of events). We also recorded whether the trial outcome was a hard outcome (ie, a clinical endpoint such as major adverse cardiovascular event or mortality) or soft outcome (ie, a surrogate marker such as change in blood pressure). For trials with hard outcomes, the number of clinical endpoint events was added to the number of serious adverse events before comparing event rates with the community population, as both endpoints and serious adverse events are likely to represent hospitalisation or deaths. We included serious adverse events and clinical endpoints from both the treatment and control arms of each trial, as most serious adverse events in the trial setting are not specifically related to the trial treatment.^23^ To confirm this, we also compared serious adverse event rates in the treatment and placebo arms.

For each participant in the community sample, we identified age and sex. We then calculated the number of all-cause emergency or urgent hospitalisations (excluding elective admissions) or deaths occurring over 3 years of follow-up. Participants were censored at death or if they deregistered from a participating practice within the 3-year period.

### Statistical analysis

Our first analysis compared the serious adverse event rate in trials of older people with the rate in standard trials, adjusting for trial characteristics. We modelled serious adverse events on older-people trial status using hierarchical Poisson regression models (with random intercept and Poisson likelihood), both unadjusted (offset by estimated person-time, which was calculated as follow-up × (number of participants – 0·5 × number of serious adverse events)) and adjusted for direct renin inhibitor trial (yes or no), comparison type (placebo, different ATC class to three-character class [eg, an RAAS drug *vs* a calcium channel blocker], or different ATC class to five-character class [eg, an angiotensin II receptor blocker *vs* an angiotensin-converting enzyme inhibitor]), phase (3 or 4), and outcome type (hard or soft). The adjusted model was the prespecified primary analysis and was used to calculate incidence rate ratios (IRRs). Models were fitted using the rstanarm package to allow fitting of the random intercept for the trials and calculation of 95% credible intervals (CrIs).

We also used Poisson regression to model the age-specific and sex-specific rate of emergency or urgent hospital admissions or death in the 3 years following initiation of RAAS drugs in SAIL. This model fitted the data well (appendix pp 1–4), and the covariates and variance–covariance matrix were exported from the SAIL secure platform to allow us to calculate the expected number of hospitalisations and deaths for each trial population, as a proxy for serious adverse events. We then calculated the ratio of expected-to-observed serious adverse events. We used the truncated normal distribution to estimate the age distribution for each trial based on the reported mean age, as well as any age cutoffs used as exclusion criteria. In a previous analysis of individual participant data (including trials with the same eligibility criteria as trials included in the current sample), the truncated normal distribution was found to accurately represent the age distribution of trials in this context.^12^ We generated 95% CrIs for the expected-to-observed ratio for each trial as follows. We obtained 10 000 samples of the intercept, age, and sex coefficients by sampling from a multivariate normal distribution where the parameters were the point estimates and variance–covariance matrix for the SAIL Poisson regression models. For each sample, we applied the coefficients to the age-sex distribution of each trial to obtain 10 000 samples from the distribution of the expected count. We then divided each of these by each of 10 000 samples from a Poisson distribution (where the parameter was the observed count) to obtain 10 000 samples representing the uncertainty distribution for the expected-to-observed ratio, which we summarised by the mean and 2·5th and 97·5th centiles.

We obtained the standardised ratio (SR) of hospitalisations and deaths in the community and serious adverse events in the trials by treating the log of the expected count (which was obtained by applying the SAIL-derived age-sex-specific rates to the age-sex distribution of each trial) as an offset term in the previously described hierarchical Poisson regression models. The first model compared standard and older-people trials. The second model further adjusted for trial characteristics.

We did three sets of sensitivity analyses. First, in view of the small number of older-people trials, we reran the regression models excluding each trial in turn to examine the sensitivity of the findings to trial characteristics. The second sensitivity analysis explored the impact of possible misclassification of the indication for RAAS treatment within the community cohort. For this, in addition to excluding participants with recent myocardial infarction or stroke (as done in the main analysis), we also excluded any participant with a previous diagnosis of diabetes, heart failure, or chronic kidney disease. We then repeated all analyses comparing trials with the community cohort. Finally, as the trial follow-up periods were shorter than the observation time of the community cohort, we repeated all analyses limiting follow-up of the community sample to the first 90 days following initial prescription (to match the median follow-up in the trials) and analysing first event only (ie, censoring at 90 days, first hospitalisation, or death, whichever happened first).

All analyses were done using R (version 3.6.1).

### Role of the funding source

The funder of the study had no role in study design, data collection, data analysis, data interpretation, or writing of the report.

## Results

**Figure 1:**
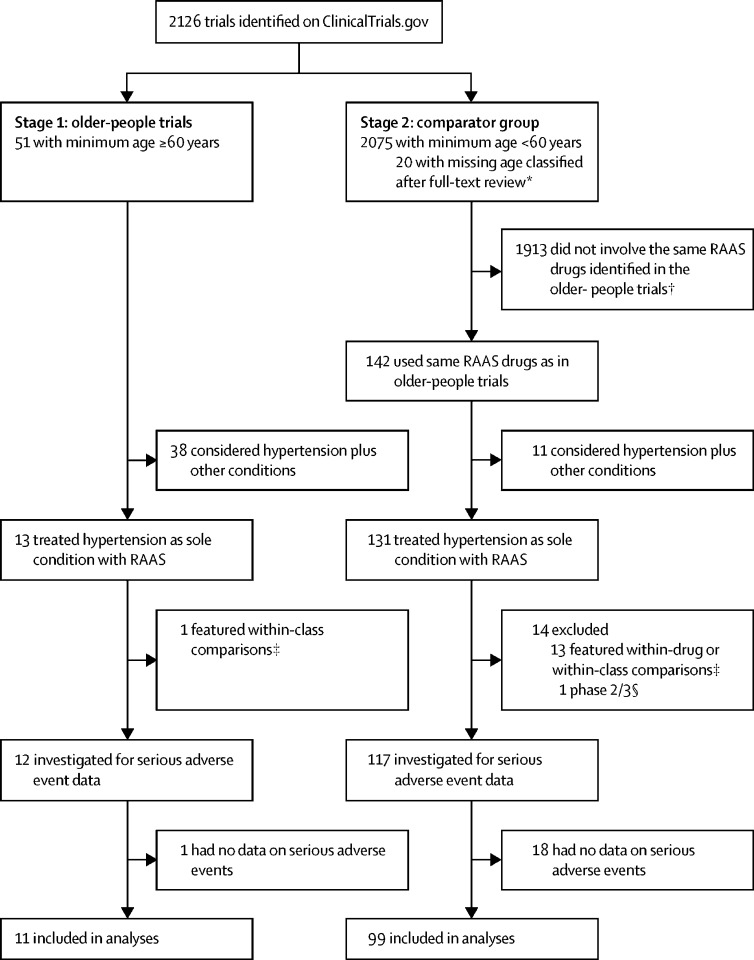
Trial inclusion ATC=Anatomic Therapeutic Chemical. RAAS=renin-angiotensin-aldosterone system. *The entry for the specific field in ClincialTrials.gov for the minimum age was missing. The full text of the trial registration was then reviewed to identify if the trial was targeted specifically at older participants. †All RAAS drugs were permitted for the selection of eligible older-people trials. Only drugs that were studied in one or more of the older-people trials (aliskiren, irbesartan, olmesartan, telmisartan, or valsartan) were selected for the comparator group of the standard trials. ‡Within-drug comparisons refers to trials where all arms included the same drug (eg, trials of different dosages or regimens). Within-class comparisons refers to trials where all arms included drugs with the same five-character ATC class (eg, drugs in WHO ATC class C09CA are all angiotensin II receptor blockers). §Excluded because none of the older-people trials were phase 2/3.

We included 110 trials, of which 11 (10%) were trials in older people and 99 (90%) were standard trials that did not focus specifically on older people (figure 1; table). Trial characteristics for all included trials, such as NCT number, trial setting, and number of participants, are available online. The median number of serious adverse events per trial was 7·5 (IQR 3·0–14·0). The median rate of serious adverse events per person per year was 0·18 (0·12–0·29) in the older-people trials and 0·11 (0·08–0·18) in the standard trials. These serious adverse event rates refer to the whole trial population, as rates were similar between treatment and control arms in any placebo-controlled trials (IRR 0·81, 95% CrI 0·59–1·08).

Before adjusting for trial characteristics, the IRR for older-people versus standard trials was 1·70 (95% CrI 1·07–2·77). After adjusting for trial drug, type of comparison, trial phase, and type of outcome, older-people trials had a higher incidence of serious adverse events than did standard trials (1·76, 1·01–3·03).

**Figure 2:**
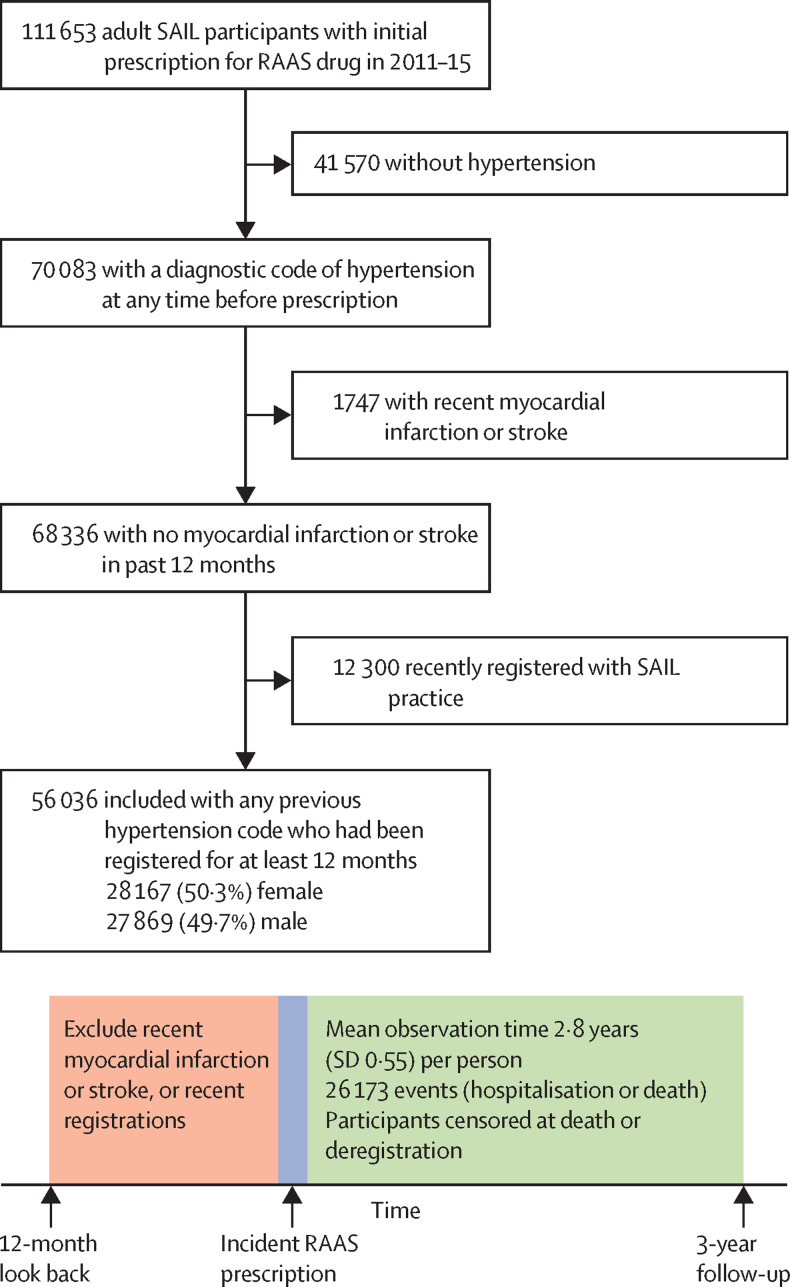
Inclusion and analysis of SAIL participants for community comparison RAAS=renin-angiotensin-aldosterone system. SAIL=Secure Anonymised Information Linkage.

**Figure 3:**
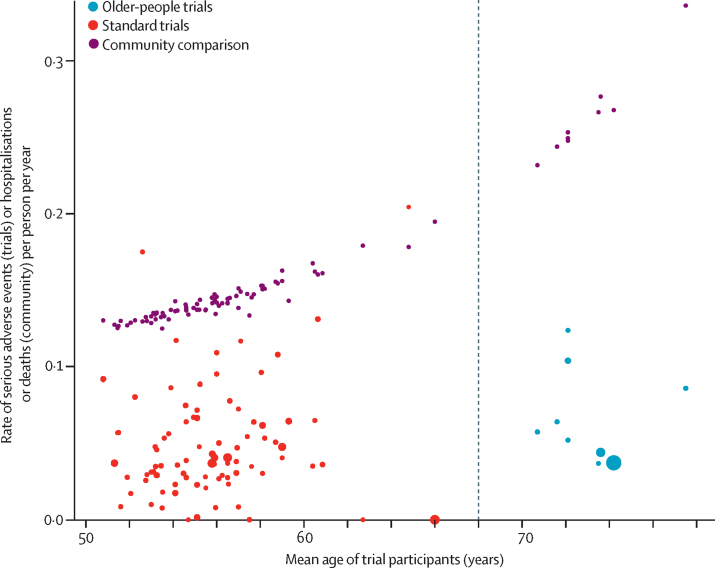
Observed versus expected serious adverse events per trial The observed rate of serious adverse events per trial is plotted at the mean age for the trial, with the sample size for each trial indicated by the size of the point. The expected number of hospitalisations and deaths for each trial, obtained by applying community all-cause non-elective hospitalisation and death rates to the age-sex distribution of each trial, is shown by the purple points. Each trial point has a community comparison point which is its pair, but lines connecting these are not shown for clarity. The vertical dashed line shows the division between standard and older-people trials.

Our SAIL community sample consisted of 56 036 individuals starting RAAS drugs in routine clinical practice, who experienced 26 173 emergency hospitalisations or deaths over the 3-year follow-up (figure 2; table). When applying community all-cause non-elective hospitalisation and death rates to the age-sex distribution of each trial, the observed rates were consistently lower than the age-adjusted and sex-adjusted expected rates (figure 3).

**Table:** Summary of included trials

		**Standard trials (n=99)**	**Older-people trials (n=11)**	**Community comparison (SAIL; n=56 036)**
Mean age or median of trial mean ages, years		55·6 (53·7–57·0)	73·1 (71·6–74·2)	60·6 (13·9)
Percentage of women		45% (40–49)	55% (52–55)	50·3%
Drug under investigation				
	Angiotensin receptor blocker	66 (67%)	8 (73%)	..
	Renin inhibitor	33 (33%)	3 (27%)	..
Comparison				
	Placebo	22 (22%)	1 (9%)	..
	Drug of different class	77 (78%)	10 (91%)	..
Phase				
	3	67 (68%)	5 (45%)	..
	4	32 (32%)	6 (55%)	..
Trial endpoint				
	Hard	1 (1%)	2 (18%)	..
	Soft	98 (99%)	9 (82%)	..
Trial sample size		722 (474–1124)	754 (388–884)	..
Trial follow-up, days		63 (56–98)	98 (56–252)	..

Data are median (IQR), mean (SD), n (%), or %. Data for each trial are available online, including data on baseline blood pressure (four [36%] older-people trials and 46 [46%] standard trials), comorbidity status (three [27%] older-people trials and 37 [37%] standard trials), and ethnicity (two [18%] older-people trials and 39 [39%] standard trials). These are not summarised in this table due to the high proportion of missing data. SAIL=Secure Anonymised Information Linkage.

**Figure 4:**
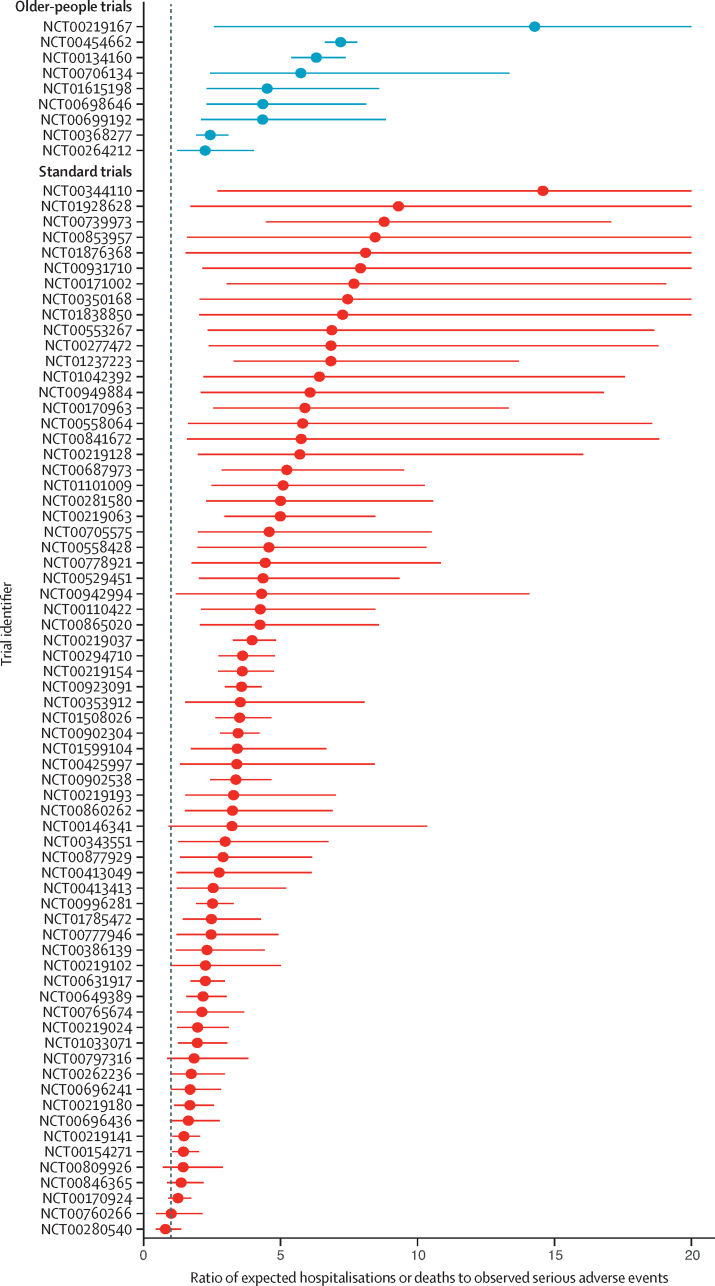
Ratio of expected-to-observed serious adverse event count Each point (with 95% CI) shows the ratio of expected all-cause non-elective hospitalisations or deaths (given the estimated age-sex distribution of each trial) to the observed serious adverse event count in each trial. Four standard trials that reported no serious adverse events are excluded from this figure as the ratio would have been infinite. Seven standard trials that reported only one serious adverse event, and thus had ratios greater than 50, are also excluded. A further 22 trials were excluded as they reported insufficient data on the age-sex distribution to calculate the expected rate.

The rate of serious adverse events was lower than the expected rate of hospitalisations and deaths given the age-sex distribution of trial participants for all but one of the trials (with this exception being a standard trial; figure 4). There was considerable heterogeneity in the calculated ratios within both the older-people trials and the standard trials. However, across all trials, the reported rate of serious adverse events was considerably lower than would be expected if the trials were representative of people with hypertension taking RAAS drugs in the community. The SR was 4·23 (95% CI 3·51–5·09) for standard trials and 4·76 (2·89–7·86) for older-people trials, indicating that hospitalisations and deaths occurred more than four times more frequently among people taking RAAS drugs in the community than serious adverse events occurred in trials. The magnitude of risk increase for serious adverse events in community patients taking RAAS did not differ when comparing older-people and standard trials (ratio of SRs 1·13, 95% CI 0·66–1·92). The results were similar after adjusting for trial drug, type of comparison, trial phase, and type of outcome (adjusted SR 4·53 [2·84–7·37] for standard trials and 4·88 [2·34–10·48] for older-people trials; ratio of SRs 1·08 [0·58–2·02]).

In the first sensitivity analysis, the effect estimates were similar when leaving out each trial in turn. In the second sensitivity analysis, the difference in serious adverse events rates between trials and the community was similar after further excluding people with diabetes, heart failure, or chronic kidney disease from the community sample to minimise the risk of misclassification of the indication for RAAS treatment (appendix pp 5–7). In the final sensitivity analysis, when limiting follow-up of the community sample to 90 days to match median follow-up time of trials, the difference in rates between trials and community remained similar (appendix pp 8–10).

## Discussion

In this analysis of trials of RAAS drugs for hypertension, trials specifically recruiting older people (minimum inclusion age ≥60 years, mean age >70 years) had a significantly higher incidence of serious adverse events than did standard trials, after adjusting for trial characteristics. This suggests that trials of older people recruit participants with a higher baseline risk of adverse health outcomes. Nonetheless, both in trials of older people and standard trials, the rate of serious adverse events was substantially lower than the expected rate based on the incidence of non-elective hospitalisation and death—which would be classed as serious adverse events in all trials—in people with hypertension being treated in the community. The difference in rates was large, with rates of hospitalisations and deaths in the community on average four times greater than the rate of serious adverse events in the trials. This suggests that, even accounting for age and sex, participants in hypertension trials and people with hypertension in the community are very different populations. These differences between trial and community populations might reflect differences in geographical or health-care settings of trials and the community cohort in Wales (although hospitalisation rates in the UK are comparable with other countries within the Organisation for Economic Co-operation and Development, suggesting that the rates observed in the community sample used in this study are similar to other settings), as well as demographic and clinical differences between the trial and community populations. These differences, in part driven by trial exclusion criteria, might include comorbidity and underlying health status, as well as other factors such as ethnicity, socioeconomic status, hypertension severity, health-care utilisation, and medication adherence. Of note, many of these factors were not reported in the included trials.

The difference in serious adverse event rates between trial and community populations was similar for older-people trials and for standard trials. This does not necessarily mean trial findings are inapplicable to community patients. Relative treatment benefits estimated in trials will often be applicable even where there are differences between trial and target populations,^24^ but net benefit can still vary because adverse events are more common in community populations, and optimal choice of drug might be affected by comorbidity and co-prescribing, which are more likely in the community. This suggests that clinical guideline developers are correct to be cautious when applying trial evidence to community populations, particularly to older, multimorbid, or frailer populations, and that this caution remains justified even when trials are deliberately targeted at older people.

While these findings suggest that trials are under-representative in terms of underlying risk of adverse health outcomes, there are two alternative explanations that could also contribute to the observed differences between trials and the community sample. First, trials might under-report the true incidence of serious adverse events. Despite reporting guidelines,^20^ there is inconsistency in how serious adverse events are reported.^25^ However, trial-recorded serious adverse events include events other than emergency hospitalisations or death, meaning that the incidence of serious adverse events in trials would be expected to be higher than the community events examined. Second, our community sample might include people taking RAAS for other indications, for whom the risk of hospitalisations and deaths might therefore be higher. For this reason, the primary analysis excluded people with recent myocardial infarction or stroke, and the sensitivity analysis excluded people with a history of diabetes, chronic kidney disease, or heart failure, with consistent findings across all analyses. Nonetheless, we cannot be certain about the true indication for starting RAAS drugs from routine data alone. Both under-reporting of serious adverse events in trials and misclassification of the community comparison might bias our estimation of the difference between trials and community samples. However, the difference between trial and community populations was large, and it is likely that the observed lower rates of serious adverse events in trial populations is accurate.

Our findings have implications for interpreting trials that specifically recruit older people. Such trials are likely to be helpful in informing treatment decisions as they more successfully recruit older people who are at higher risk of serious adverse events than do standard trials, thus capturing some of the increased risk of this population. However, concerns about trial representativeness are still well founded, as suggested by the observed difference between serious adverse events in trials and hospitalisation and death rates in the community. We observed that this difference was similar for both trials of older people and standard trials, suggesting that they were similarly under-representative. This suggests that trials focusing on older people present only part of the solution to informing treatment decisions for older people, particularly those at higher risk of adverse health outcomes, such as people living with frailty.

The higher rate of hospitalisations and deaths in the community population has some important implications for managing hypertension in older people. First, this finding is likely to reflect a higher prevalence and severity of frailty in community populations compared with trials, which might modify the relationship between hypertension and cardiovascular risk.^4^ We previously showed, in an individual-level participant data analysis, that frailty is associated with increased risk of serious adverse events in trials.^13^ Furthermore, frailty in participants in cardiovascular trials is associated with adverse cardiovascular outcomes independently of traditional risk factors.^26^ While frailty has been shown to be present in participants of trials for hypertension in older people,^8^, ^9^ frailty in these trial participants is thought to be less severe than frailty in people in the community.^2^ People living with severe frailty are likely to be excluded from clinical trials; however, such individuals are commonly prescribed the trial medications in routine clinical practice, often in the context of polypharmacy. The applicability of trial evidence to a broader population, even in the case of trials recruiting older people, needs careful consideration. It is also likely that trial evidence is insufficient to inform treatment decisions in some patient groups, such as people living with severe frailty.

Second, the difference in serious adverse events between trial and community populations could affect the net benefit of treatment when used in routine clinical practice.^27^ For example, higher competing mortality risks in the community might mean that benefits in terms of absolute treatment effects (based on trial participants) are overestimated.^28^ Also, if drug-related serious adverse events were more common in the community (eg, among people living with frailty), this could reduce the net benefit of treatment.^29^, ^30^ For example, even if drugs reduce cardiovascular outcomes, net benefit might decrease if serious adverse event rates increase rapidly with age. Quantifying net benefit would require analysis of differential treatment effectiveness and also treatment-related serious adverse events, neither of which are possible with the data from this study. However, our findings do indicate that clinicians and guideline developers should be cautious when applying trial estimates of benefit to the wider population.

Strengths of this study include a systematic identification of registered trials. With our search of a trial register, as well as a manual search of clinical study reports, we were able to include both published and unpublished trials, limiting publication bias. Restricting our search to ClincalTrials.gov might have resulted in a small proportion of studies not being included in our investigation. However, ClinicalTrials.gov is the largest international trial registry and trial preregistration is required both for publication in high-impact journals and for trial results to qualify as evidence for regulatory agencies such as the US Food and Drug Administration.^19^ Moreover, it provided a single sampling frame from which we could draw all older-people and standard trials. Limiting our search to trials started from 1999 onwards ensured that trials were conducted in a similar time period to the community comparison, and during a period in which trial registration has become increasingly commonplace; however, this will have led to the exclusion of earlier trials, including some of commonly prescribed RAAS drugs such as angiotensin-converting enzyme inhibitors. The systematic comparison of serious adverse event rates in trials with hospitalisation and death rates in the community for people with hypertension is novel and builds upon previous studies of trial representativeness by comparing actual health-related outcomes rather than exclusion criteria. Nonetheless, comparing serious adverse events to hospitalisations and deaths is not an exact like-for-like comparison. Serious adverse events are based on a broader definition that includes events perceived to be life threatening as well as events leading to impairment or disability, which might not necessarily result in hospitalisation. However, hospitalisations and deaths are, by definition, serious adverse events, and so any bias is very likely towards underestimating the difference between trial and community rates. Trial data were reported inconsistently, and for some trials we had to estimate the observation time in the trial (based on follow-up length and the serious adverse event rate). Also, as we have highlighted above, we were not able to verify the indication for RAAS drugs in the community. While we excluded participants with recent events that would be alternative indications, there might be some participants in our sample who were prescribed RAAS drugs for reasons other than hypertension. Finally, this study focused on RAAS drugs for hypertension, and the findings might not necessarily be generalisable to other drugs or indications, particularly as serious adverse event rates for angiotensin receptor blockers might be lower than for other antihypertensives.^29^

In conclusion, our study shows that participants in hypertension trials experience substantially lower rates of serious adverse health outcomes than do people with hypertension treated with similar drugs in the community. Our work suggests that assessment of the rate of serious adverse events in trials, when compared with the expected rate from representative target populations, could be a useful metric of trial representativeness. Our findings also show that the problem of under-representativeness is not sufficiently resolved by recruiting older people to trials, as both older-people trials and standard trials were under-representative in terms of serious adverse events. This observation emphasises the need for developing approaches to trial design and execution that enable older people living with frailty to become trial participants.

## Data sharing

All trial data and analysis code are available on Github. Aggregate data from SAIL, along with all model parameters, are available in the same repository and summarised in the appendix (pp 1–4).

## Declaration of interests

We declare no competing interests.
