# Supplementary material for: Observed and expected serious adverse event rates in randomised clinical trials for hypertension: an observational study comparing trials that do and do not focus on older people
Source: Lancet Healthy Longev. 2021 Jul;2(7):e398–406. doi: 10.1016/S2666-7568(21)00092-1 (PMC8245327; doi:10.1016/S2666-7568(21)00092-1)

THE LANCET
Healthy Longevity

Supplementary appendix

This appendix formed part of the original submission and has been peer reviewed. We post it as supplied by the authors.

Supplement to: Hanlon P, Corcoran N, Rughani G, et al. Observed and expected serious adverse event rates in randomised clinical trials for hypertension: an observational study comparing trials that do and do not focus on older people. *Lancet Healthy Longev* 2021; published online June 10. https://doi.org/10.1016/S2666-7568(21)00092-1.

Model of event rates in SAIL

The plot below shows the model predictions for the rates of all-cause hospitalisation or death among people with hypertension starting RAAS drugs, identified from the SAIL databank. The circles indicate the actual observed event rate (size indicating total number of people in the denominator). Model coefficients, variance covariance matrix, and aggregate data on observed events is also shown below.

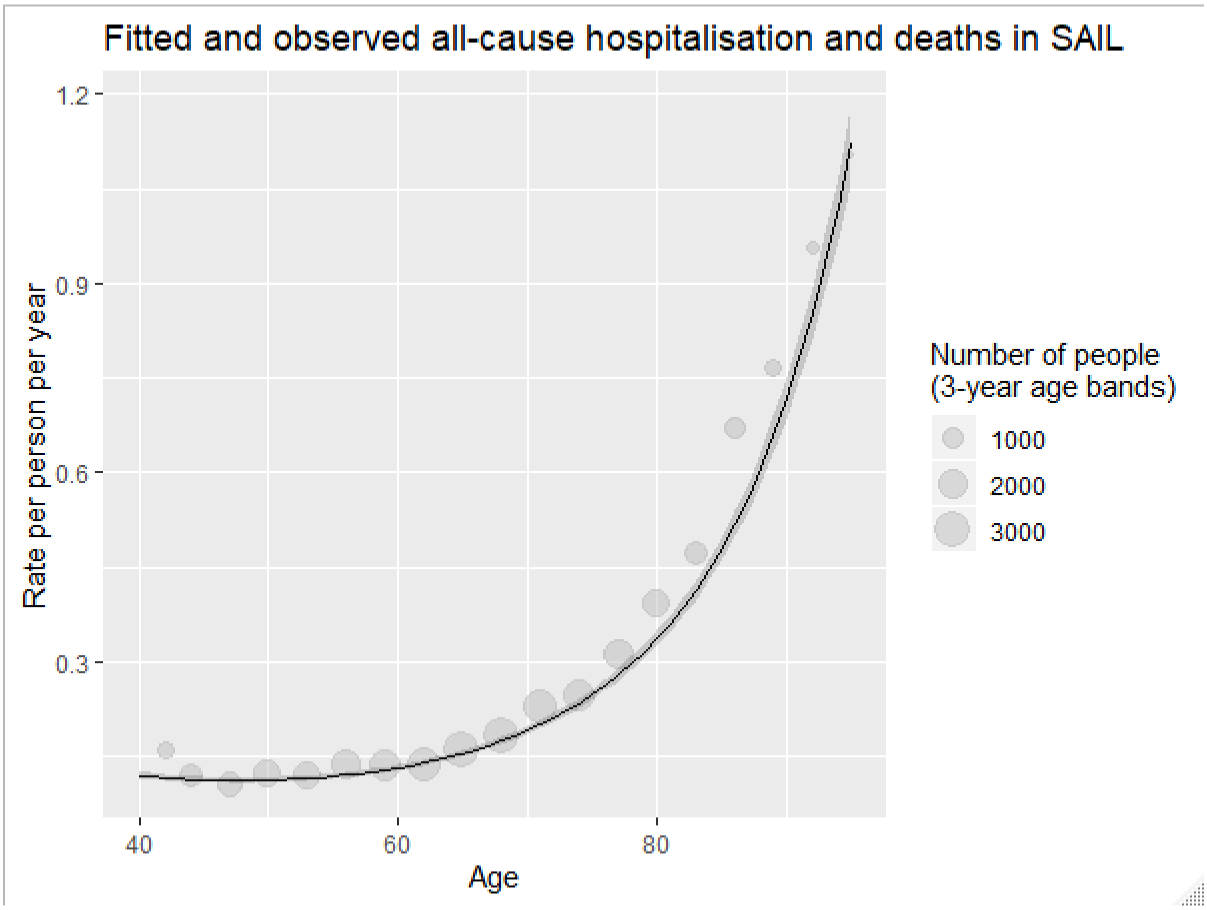

Model covariates	Coefficients
(Intercept)	-2.68478
I((age/100)^0.5)	-10.589
I((age/100)^2)	8.250124
GNDR_CD	-0.01925

	X.Intercept.	I..age.100..0.5.	I..age.100..2.	GNDR_CD
(Intercept)	0.047022	-0.07699	0.034684	-0.0004
I((age/100)^0.5)	-0.07699	0.128211	-0.05876	0.000211
I((age/100)^2)	0.034684	-0.05876	0.028355	-0.00019
GNDR_CD	-0.0004	0.000211	-0.00019	0.000208

Age	Sex	Number of people	Observation time (days)	Number of events
41	Men	390	417139	119
41	Women	277	294452	114
42	Men	446	473972	151
42	Women	319	343265	103
43	Men	489	522724	147
43	Women	356	377974	98
44	Men	500	535892	138
44	Women	445	476477	123
45	Men	596	638588	179
45	Women	467	500202	149
46	Men	649	688950	152
46	Women	497	533494	168
47	Men	652	694617	196
47	Women	554	596903	186
48	Men	726	774728	145
48	Women	610	654652	224
49	Men	693	736943	191
49	Women	652	693987	242
50	Men	879	937158	231
50	Women	671	720828	168
51	Men	815	868917	211
51	Women	709	755272	223
52	Men	785	837952	189
52	Women	670	724754	239
53	Men	806	856686	187
53	Women	641	683241	196
54	Men	771	820004	206
54	Women	645	691361	175
55	Men	717	757960	215
55	Women	589	624697	199
56	Men	688	728730	218
56	Women	521	553117	156
57	Men	659	700718	246
57	Women	597	633215	189
58	Men	644	686705	198

58	Women	554	590531	184
59	Men	631	674282	193
59	Women	528	561251	139
60	Men	681	722785	233
60	Women	567	604467	224
61	Men	698	740980	230
61	Women	604	642017	190
62	Men	678	716428	227
62	Women	601	643531	194
63	Men	773	820026	319
63	Women	634	667869	208
64	Men	792	838975	307
64	Women	766	816205	313
65	Men	796	847980	251
65	Women	755	799546	275
66	Men	747	789209	291
66	Women	785	839156	330
67	Men	711	749821	359
67	Women	794	846154	347
68	Men	716	751985	392
68	Women	789	837415	286
69	Men	575	602079	291
69	Women	672	711673	267
70	Men	605	638581	299
70	Women	706	747213	363
71	Men	525	552015	237
71	Women	627	663586	291
72	Men	525	548681	315
72	Women	624	654294	447
73	Men	460	480175	273
73	Women	595	622643	273
74	Men	410	427359	278
74	Women	627	663350	324
75	Men	395	410979	239
75	Women	600	628250	448
76	Men	405	421201	290
76	Women	575	606504	366
77	Men	336	345192	270
77	Women	502	525395	305
78	Men	324	321074	284
78	Women	538	550622	385
79	Men	290	295787	269
79	Women	445	460290	345
80	Men	263	265462	239

80	Women	423	428177	344
81	Men	239	237115	247
81	Women	414	418528	371
82	Men	215	207956	228
82	Women	355	364512	294
83	Men	208	202901	214
83	Women	368	357712	366
84	Men	146	145559	168
84	Women	315	309936	353
85	Men	141	129293	168
85	Women	259	245139	290
86	Men	125	114806	187
86	Women	217	206338	324
87	Men	103	96247	160
87	Women	199	186113	270
88	Men	96	88523	146
88	Women	181	169371	215
89	Men	77	68398	100
89	Women	164	140754	268
90	Men	56	44569	100
90	Women	145	120661	219
91	Men	30	24306	42
91	Women	103	86980	199
92	Men	27	21511	42
92	Women	92	75995	146
93	Men	15	10636	30
93	Women	75	59225	125
94	Men	22	15909	56
94	Women	46	33658	64
95	Men	17	13192	29
95	Women	45	33039	66

Sensitivity analysis 1

The analysis presented in the main text compared SAE rates in RAAS trials for hypertension to rates of all-cause hospitalisation and death in people with hypertension in the community starting RAAS drugs, after excluding people with recent stroke or myocardial infarction. This sensitivity analysis repeats this same comparison after also excluding people with heart failure, diabetes mellitus, or chronic kidney disease from the community comparison.

	Standardised ratio of community rate versus trial rate for older people trials	Standardised ratio of community rate versus trial rate for standard trials	Ratio of standardised ratios (older:standard)
Main analysis			
Unadjusted[a]	4.76 (2.89-7.86)	4.23 (3.51-5.09)	1.13 (0.66-1.92)
Adjusted[b]	4.88 (2.34-10.48)	4.53 (2.84-7.37)	1.08 (0.58-2.02)
Sensitivity analysis			
Unadjusted[a]	4.00 (2.44-6.67)	3.45 (2.78-4.17)	1.19 (0.68-2.08)
Adjusted[b]	4.17 (1.96-8.33)	3.57 (2.27-5.56)	1.15 (0.61-2.13)

[a] offset by estimated follow-up time
[b] additionally adjusted for direct renin inhibitor trial (yes/no), comparison type (placebo, different ATC class to 3-character, different ATC class to 5-character), phase (3 or 4) and outcome type (hard or soft).

Observed and expected event rates: sensitivity analysis

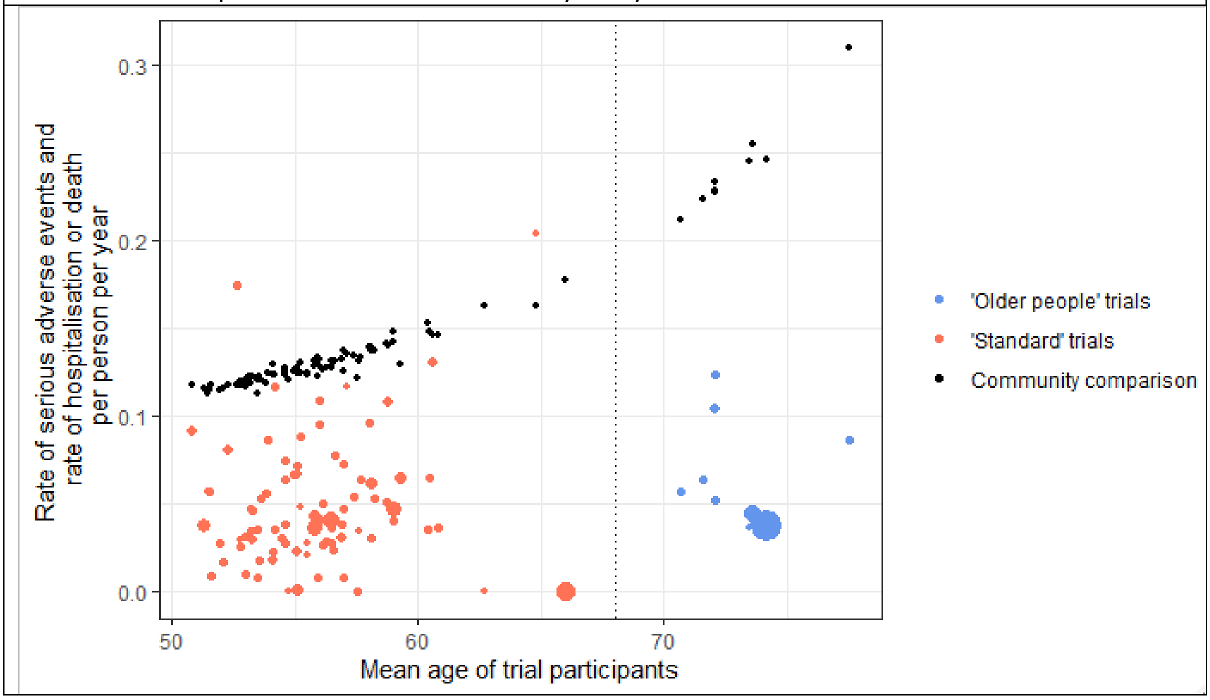

Ratio of observed to expected: sensitivity analysis

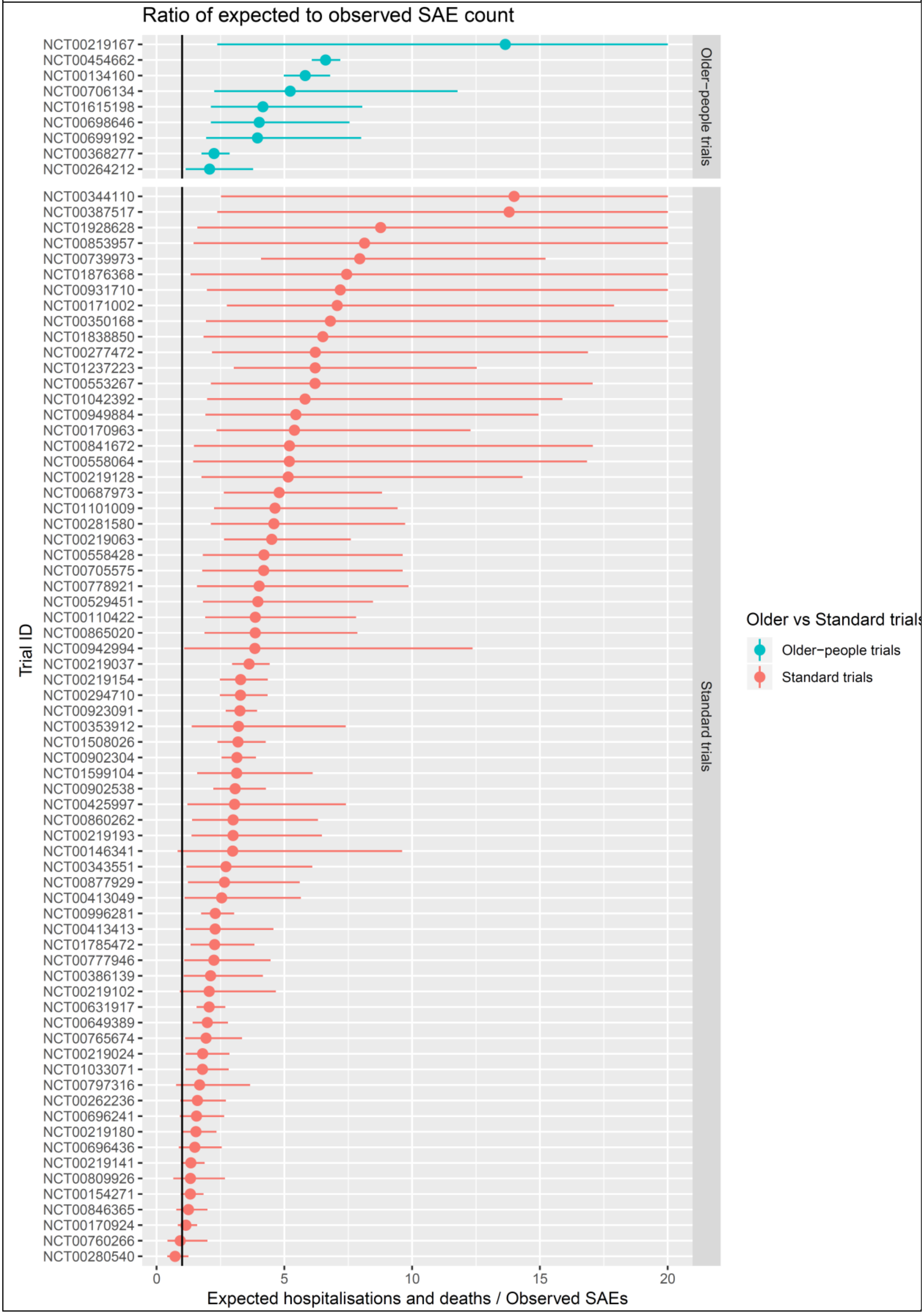

Sensitivity analysis 2

This sensitivity analysis was carried out post-hoc. As the follow-up period for the trials was shorter than the community comparison, this analysis limited follow-up time in the community to 90 days. Participants were censored at first event (hospitalisation or death) or 90-days post starting RAAS treatment, whichever occurred first. The findings are shown below.

	Standardised ratio of community rate versus trial rate for older people trials	Standardised ratio of community rate versus trial rate for standard trials	Ratio of standardised ratios (older:standard)
Main analysis			
Unadjusted[a]	4.76 (2.89-7.86)	4.23 (3.51-5.09)	1.13 (0.66-1.92)
Adjusted[b]	4.88 (2.34-10.48)	4.53 (2.84-7.37)	1.08 (0.58-2.02)
Sensitivity analysis			
Unadjusted[a]	3.70 (2.17-6.25)	3.33 (2.78-4.00)	1.12 (0.64-2.00)
Adjusted[b]	3.85 (1.85-8.33)	3.57 (2.27-5.88)	1.06 (0.58-1.92)

[a] offset by estimated follow-up time
[b] additionally adjusted for direct renin inhibitor trial (yes/no), comparison type (placebo, different ATC class to 3-character, different ATC class to 5-character), phase (3 or 4) and outcome type (hard or soft).

Observed and expected event rates: sensitivity analysis 2

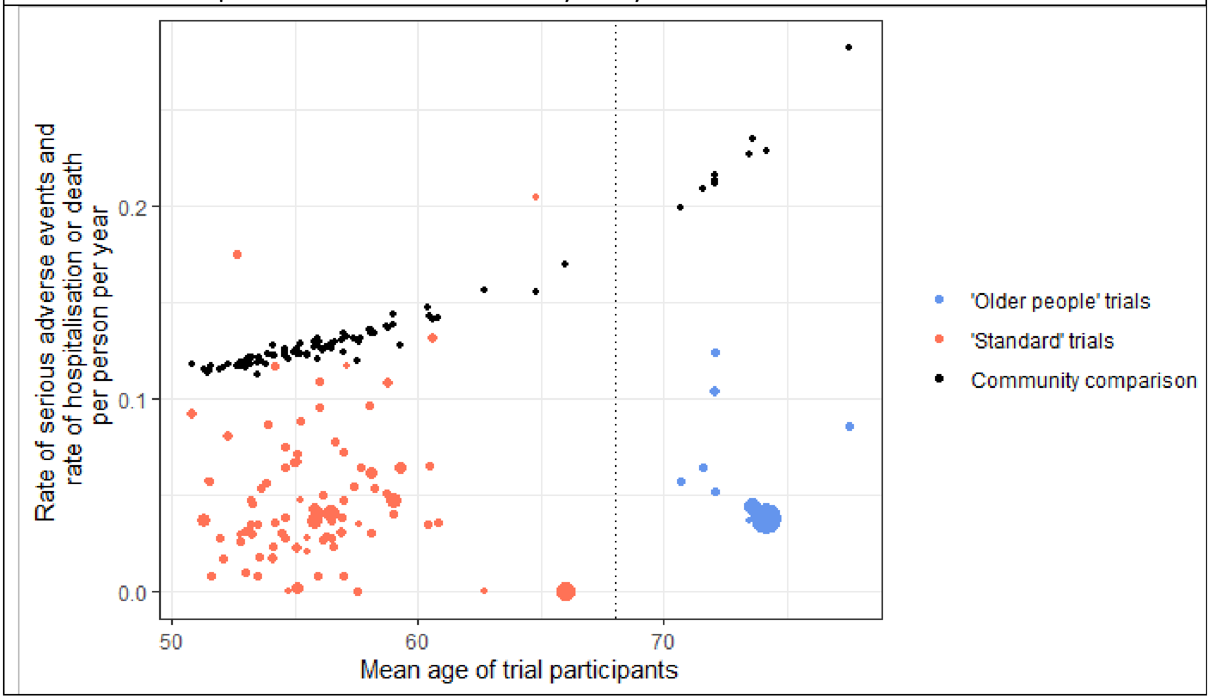

Ratio of observed to expected: sensitivity analysis 2

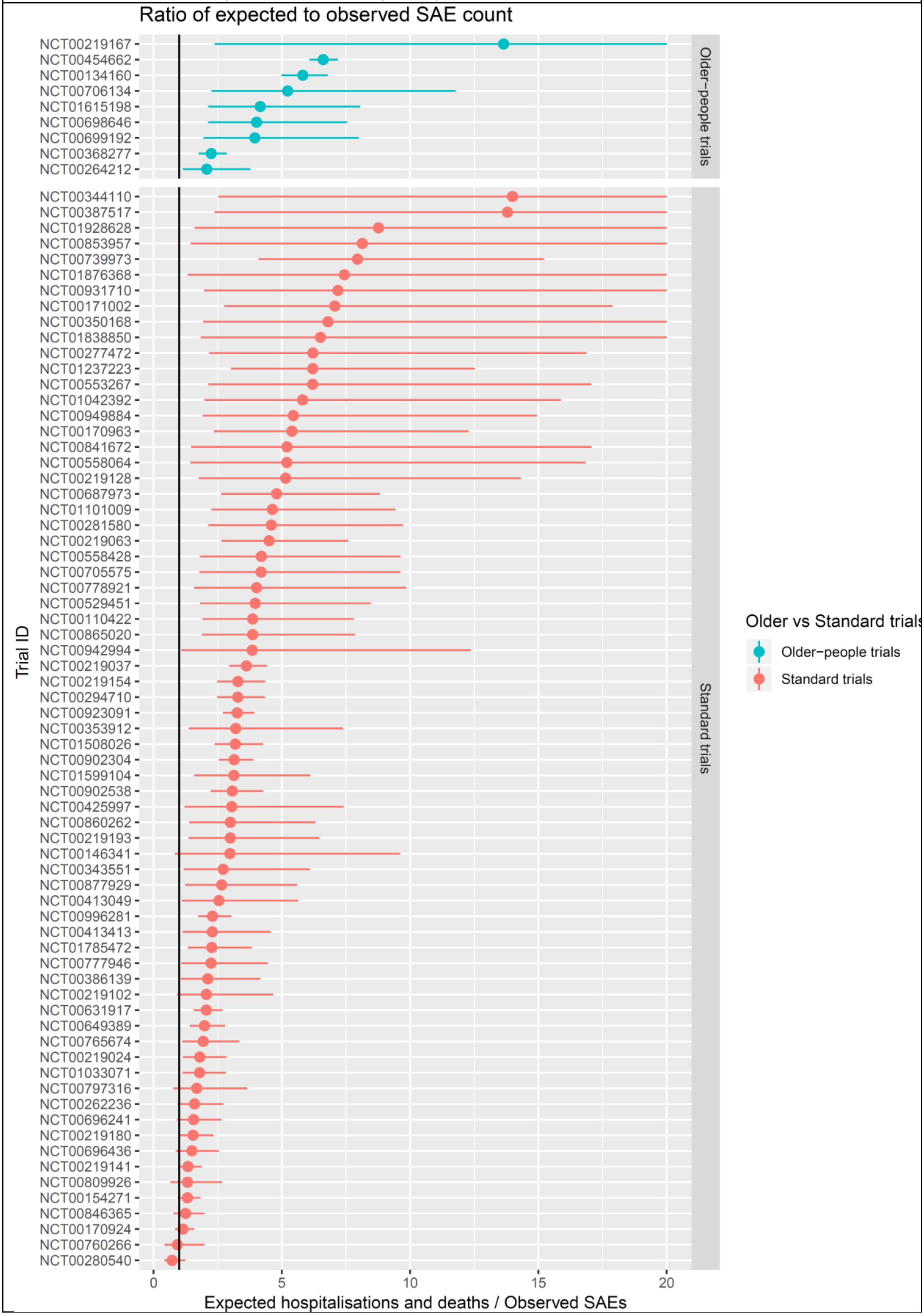

Supplement: Supplementary appendix [file mmc1.pdf]
